# Is bigger really better? Relative and absolute body size influence individual growth rate under competition

**DOI:** 10.1002/ece3.2978

**Published:** 2017-04-17

**Authors:** Josh Van Buskirk, Eva Cereghetti, Julia S. Hess

**Affiliations:** ^1^ Evolutionary Biology & Environmental StudiesUniversity of ZürichZürichSwitzerland

**Keywords:** amphibian, body size, competition, growth rate, size variability

## Abstract

Models suggest that the mechanism of competition can influence the growth advantage associated with being large (in absolute body size or relative to other individuals in the population). Large size is advantageous under interference, but disadvantageous under exploitative competition. We addressed this prediction in a laboratory experiment on *Rana temporaria* tadpoles competing for limited food. There were 166 target individuals spanning a 10‐fold range in body mass reared for 3 days with three other individuals that were either the same size, half as large, or twice as large as the target. Relative growth rate (proportion per day) declined with size, and absolute growth rate (mass per day) reached a peak at intermediate size and declined thereafter. Tadpoles grew slowly if they were large relative to their competitors, although relative body size was less important than absolute size. As a result, size variation declined in groups that were initially composed of individuals of variable size. Thus, bigger was not better under exploitative competition. Our results help connect individual‐level behavior with individual growth and the size distribution of the population.

## 1 Introduction

The growth rate of an individual organism has far‐reaching ecological and evolutionary implications, and this has prompted interest in factors that influence growth (Arendt, 1997; Dmitriew, 2011; Shelton et al., 2013). Environmental conditions such as temperature, resource availability, and the presence of competitors are known to be important, but growth rate can also depend on the size of the individual organism (Brown, 1946; Grime & Hunt, 1975; Peacor & Pfister, 2006; Rees et al., 2010; Werner, 1986; Wilbur & Collins, 1973). However, the scaling of growth with body size is modified by the environment, and in some cases the impact can be dramatic. For example, the relative (or specific) growth rate of fish reared in the laboratory tends to decline with body size, but the direction of this relationship can be completely reversed if fish are placed in social groups. Many species of fish establish dominance hierarchies, which usually reflect the size hierarchy, and socially dominant individuals grow relatively rapidly (Abbott & Dill, 1989; Brown, 1946; Grobler & Wood, 2013; Karplus, Popper, & Goldan, 2000; Symons, 1971). As a consequence, the growth rate of a fish depends not only on its body size, but also on its size relative to the other individuals with which it interacts.

General models of individual growth rate suggest that the implications of absolute and relative body size depend on the mechanism of interaction among individuals. Interference competition enables large individuals to expropriate a disproportionate share of available resources, whereas exploitative competition is more likely to disadvantage large individuals because of their higher metabolic costs (Peacor, Bence, & Pfister, 2007a; Persson, 1985; Uchmanski, 1985; Werner, 1994; Yodzis & Innes, 1992). In fish—for which interference is often important—these models correctly predict that growth should be associated positively with body size relative to others in the group (e.g., Abbott & Dill, 1989; Brown, 1946; Karplus et al., 2000). Several studies of amphibian larvae also show that larger individuals enjoy a growth advantage in situations where interference is prevalent. For example, Smith (1990), Ziemba and Collins (1999), and Doyle, Nolan, and Whiteman (2010) worked with *Ambystoma* salamander larvae and observed aggressive or cannibalistic behavior directed toward relatively small individuals. Two laboratory experiments demonstrating an advantage of relatively large body size in anuran tadpoles (Lea, Dyson, & Halliday, 2002; Woodward, 1987) may also have involved interference competition. In anurans, interference competition is mediated by *Anurafeca richardsi*, a protozoan parasite that establishes in laboratory experiments (Baker, Beebee, & Ragan, 1999; Richards, 1962; Steinwascher, 1978; Wong, Griffiths, & Beebee, 2000). Some time is required for infections of *A. richardsi* to grow large enough to suppress growth, and smaller tadpoles are affected most severely. The studies of Woodward (1987) and Lea et al. (2002) extended for the entire larval stage, so there would have been ample time for *A. richardsi* to proliferate.

There are fewer studies of the consequences of relative body size under exploitative completion in animals. Anuran larvae in Werner's (1994) study competed primarily for resources, and smaller individuals of two species were superior competitors in terms of tolerance of competition and impact on other size classes. Werner argued that this arose from the size scaling of somatic and trophic structures in amphibian larvae (Wassersug, 1975; Wassersug & Hoff, 1979), which implies that smaller individuals are relatively efficient especially under resource limitation. This is consistent with theory (Brown, Gillooly, Allen, Savage, & West, 2004; Werner & Gilliam, 1984; Yodzis & Innes, 1992). Thus, theory and empirical results suggest that the consequences of large relative body size depend on the mechanism of interaction among competing individuals.

We addressed this idea by establishing groups of *Rana temporaria* tadpoles that competed primarily by exploitative competition. The experiment measured simultaneously the influence of absolute body size and relative size on growth rate, and our prediction was that growth declines as tadpoles become larger in absolute terms and relative to others in the group. A key feature of the design is that the laboratory growth assay lasted only 3 days. This is short enough that growth rate could be associated with the absolute size and relative size of a specific target individual, and too short for appreciable infections of *A. richardsi* to develop. Our results contribute toward a mechanistic understanding of variation in individual growth rate and the genesis of size variation within populations (Magnuson, 1962; Peacor, Schiesari, & Werner, 2007b; Pfister & Stevens, 2002; Weiner & Thomas, 1986).

## 2 Methods

The experiment had a three‐by‐three incomplete factorial design with 26 replicates. Each replicate consisted of seven treatments in which a target tadpole of *R. temporaria* was reared in the presence of three other nontarget competitors. The two experimental factors were the absolute size of the target animal (small, medium, large) and the size of the competitors relative to the target (smaller, same size, larger). We matched each target with three other tadpoles to ensure that the effect of competitors was relatively strong. Two treatments were not implemented: The smallest size class could not be matched with even smaller individuals and the largest size class could not be matched with larger individuals.

The tadpoles were reared in opaque polypropylene containers (20 × 12 cm) filled with 1 L aged tap water and placed on three shelves in an indoor laboratory. Containers were grouped into blocks by shelf and spatial proximity within shelves, and treatments were assigned at random within blocks. Artificial light was provided 14 hr per day, and some natural light came through windows along one wall. The temperature of the room was between 18 and 21°C.

The experimental animals came from six clutches of *R. temporaria* collected 9 km south of Bonaduz, Switzerland. We generated variation in body size by rearing tadpoles for 2 weeks at three densities in outdoor plastic tubs (80 L; six tubs each with 53, 161, and 429 tadpoles/m^2^). The 18 tubs were stocked 3 days after hatching on 15 April 2015, when tadpoles weighed 14.2 mg and were at Gosner (1960) stage 23.8. Tadpoles were fed twice a week on 2 g rabbit pellet food per tub.

**Figure 1:**
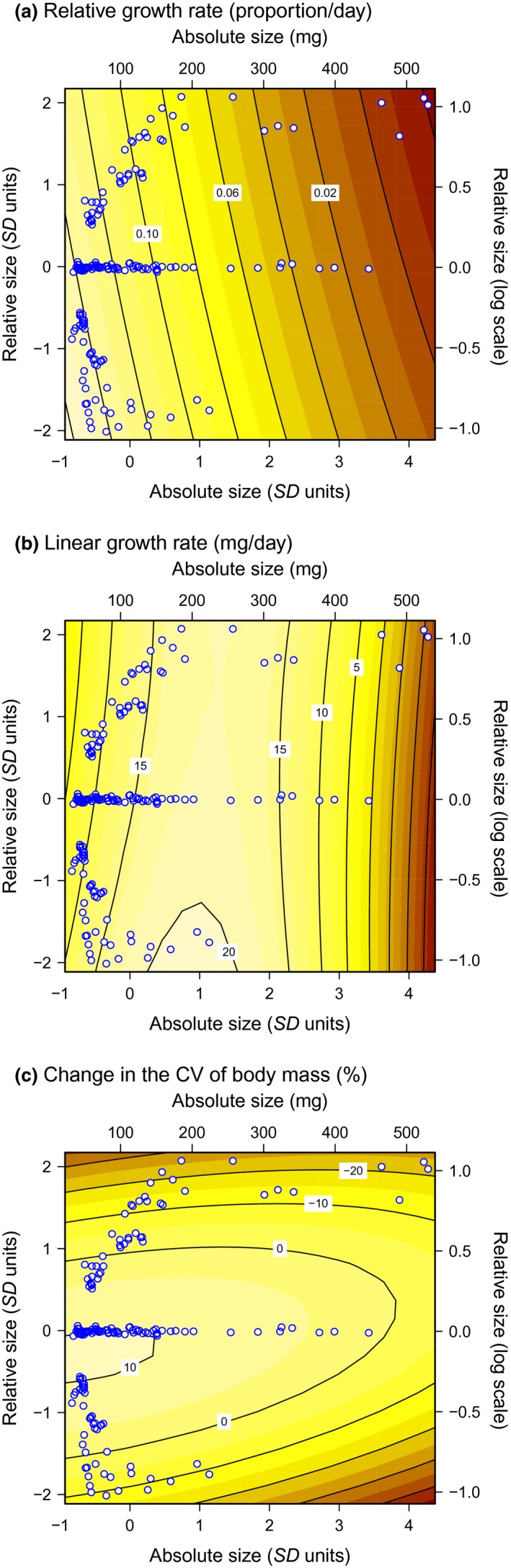
Fitted surfaces depicting growth rate and size variation as a function of the absolute and relative body size of the target tadpole compared with its three competitors. The contour surface in (a) represents daily proportional growth rate, in (b) the linear growth rate, and in (c) the change in the coefficient of variation (CV) in mass of the four tadpoles sharing the container. The three panels correspond to the statistical models in Table 1. Points represent the sizes of the 166 target individuals. Positive relative size indicates that the target individual was larger than its competitors. The axes are shown on the original scale (top, right) and the standardized scale on which the analysis was performed (bottom, left; mean = 0, *SD* = 1)

We conducted three rounds of the experiment over 3 weeks, completing a total of 26 replicates. On each round, 300 tadpoles from the outdoor tubs were weighed and classified into small, medium, and large categories. Developmental stages were not recorded, but initial masses indicate that Gosner (1960) stages ranged from 26 to about 35, well before *R. temporaria* initiate premetamorphic weight loss (Van Buskirk, 2002). We allocated tadpoles to experimental containers haphazardly, while ensuring that the three nontarget competitors were of similar size and, as far as possible, maintaining a constant size ratio between larger and smaller size classes. Target tadpoles were photographed in lateral view for later identification, except in the three same‐size treatments, where we selected the target individual at random when the experiment ended. We did this because there was almost no variation in initial mass among the four tadpoles placed together in the same‐size treatments: the target:nontarget ratio averaged 1.000 ± 0.014 *SD*. In treatments with larger competitors, target individuals were about half as large as nontargets (ratio: 0.528 ± 0.116 *SD*), and in treatments with smaller competitors, they were about twice as large as nontargets (ratio 1.947 ± 0.477 *SD*). Figure 1 shows the absolute and relative body sizes of every target tadpole.

The three densities in the outdoor rearing phase did not contribute target tadpoles equally often. High‐density rearing tubs produced 84% of target individuals for the treatment with larger competitors, 62% of targets for the same‐size treatment, and 45% of targets for the treatment with smaller competitors.

Each round of the experiment lasted 3 days. Tadpoles were fed every day on ground rabbit food equivalent to 8% of the combined mass of the four animals in the container. Anuran growth rate under this quantity of food is about 15%–40% lower than under ad libitum quantities (Anholt, Werner, & Skelly, 2000; Muenst, 2015). After 3 days, tadpoles were removed from the experiment, weighed, and photographed for identification. Working with live wet weight, which was necessary in order to measure growth during the experiment, meant that we could not eliminate individual variation in the contents of the bladder or stomach. Individuals were never reused in subsequent experimental rounds.

We calculated growth rate of the target tadpole in two ways. Relative growth rate was the proportional daily growth increment: (ln(*m*_2_) − ln(*m*_1_))/3 days, where *m*_1_ and *m*_2_ are the mass of the target tadpole on the first and last day, respectively. This measure assumes that growth follows an exponential model, which is appropriate for anurans over intermediate durations of the early and middle larval stage (Mansano, De Stefani, Pereira, Nascimento, & Macente, 2014; Muenst, 2015; Van Buskirk, 2002; Wilbur & Collins, 1973). Linear growth rate was the daily growth increment in mg: (*m*_2_ − *m*_1_)/3 days. This measure assumes that growth is linear, which is probably appropriate over the very brief duration of this experiment.

### 2.1 Statistical analyses

Of the 182 sets of tadpoles in the experiment, 166 remained for analysis after discarding containers in which any of the four individuals died. Our main aim was to estimate the relationship between absolute and relative body size and growth rate; in addition, we explored the consequences of growth variation for the population size distribution. This was performed with three mixed‐effects linear models, one for each measure of growth and one for the change in the coefficient of variation (CV) in size of the four individuals within the container over the 3 days of the experiment (CV_final_ − CV_initial_). In all cases, the model included fixed effects of absolute size (linear and quadratic terms), relative size (linear and quadratic terms), and the interaction between absolute and relative size. Absolute body size was the mass at the beginning of the experiment (mg). Relative size was the difference in mass between the target tadpole and the average of the nontarget tadpoles on a logarithmic scale: ln(*m*_1_) − ln(${\bar{m}}_{nt}$), where ${\bar{m}}_{nt}$ is the average mass of the three nontarget tadpoles on the first day and *m*_1_ is as defined above. Relative size had a value of 0 if the target and nontarget tadpoles weighed the same, a value of +1 if the target was 2.7‐times larger than the nontargets, and a value of −1 if the nontargets were 2.7‐times larger than the target. Both absolute and relative size were standardized prior to analysis (mean = 0, *SD* = 1) to facilitate interpretation of the model coefficients. The random effect was block, which included variation among the three experimental rounds and among spatial positions within the room. We fitted the models using REML with the lmer function in R 3.3.0 (Bates, Maechler, Bolker, & Walker, 2015). Significance of fixed effects was evaluated by examining their profile likelihood confidence intervals (Venzon & Moolgavkar, 1988), and the importance of block was judged using likelihood ratio tests.

## 3 Results

For both measures of growth rate, the influence of absolute body size was much greater than the influence of relative body size (Table 1). Relative growth declined linearly with absolute size: The smallest tadpoles (<100 mg) grew 12%–15% per day and the largest (>400 mg) did not grow at all (Figure 1a). Linear growth rate showed a quadratic relationship against absolute size, climbing from 10 mg/day in small tadpoles toward a peak of 18 mg/day when tadpoles weighed about 200 mg and declining thereafter (Figure 1b). Growth was negatively associated with relative body size in both models. That is, target tadpoles grew somewhat more slowly if they were larger than their competitors, but this effect was only about 20% as large as that of absolute size (Table 1).

**Table 1:** Summary of mixed‐effects models estimating the linear and quadratic effects of absolute body size (mass in mg) and relative size on growth rate and the change in size variation of *Rana temporaria* tadpoles

Fixed effect	Relative growth rate (proportion/day)			Linear growth rate (mg/day)			Change in CV body mass (%)		
	Coefficient	*SE*	95% CI	Coefficient	*SE*	95% CI	Coefficient	*SE*	95% CI
Intercept	**0.1117**	**0.0063**	**(0.0994, 0.1240)**	**14.58**	**0.859**	**(12.92, 16.24)**	**10.02**	**1.310**	**(7.483, 12.56)**
Absolute size	**−0.0347**	**0.0084**	**(−0.0512, −0.0185)**	**7.289**	**1.157**	**(5.050, 9.528)**	−0.287	1.754	(−3.546, 3.248)
Relative size	−0.0082	0.0043	(−0.0163, 0.0002)	−**1.484**	**0.597**	**(**−**2.640,** −**0.329)**	−**3.429**	**0.901**	**(**−**5.218,** −**1.721)**
(Absolute size)^2	0.0016	0.0038	(−0.0056, 0.0090)	−**3.307**	**0.529**	**(**−**4.330,** −**2.283)**	−0.649	0.801	(−2.224, 0.886)
(Relative size)^2	0.0004	0.0038	(−0.0068, 0.0078)	0.239	0.533	(−0.793, 1.271)	−**7.327**	**0.807**	**(**−**8.858,** −**5.724)**
Absolute size × Relative size	0.0002	0.0058	(−0.0112, 0.0112)	0.651	0.812	(−0.921, 2.222)	1.966	1.231	(−0.429, 4.352)

Growth was measured as the proportional daily change in mass or the linear change; change in size variation was the difference between final and initial coefficient of variation (CV) among the four tadpoles. Absolute and relative size were standardized to a mean of 0 and *SD* of 1 before analysis. Bold text emphasizes effects for which the 95% confidence interval (CI) did not include zero.

Relative body size strongly influenced the change in size variation within containers (Table 1). There was little change in the CV of mass in same‐size treatments, except for a slight increase among the smallest tadpoles (Figure 1c). However, CV decreased sharply when the target tadpole was either smaller or larger than its competitors. The size distributions within containers converged over the 3 days because relatively small tadpoles grew rapidly and large tadpoles grew slowly. Variation among blocks was not important in any of the three models (likelihood ratio tests: relative growth, LR = .16, *df* = 1, *p *=* *.69; linear growth: LR = 0; change in CV: LR = 0).

## 4 Discussion

This experiment held constant a variety of factors known to influence the growth of amphibian larvae, such as temperature, the number of competitors, predation risk, and the quantity and quality of resources. Under these controlled conditions, absolute body size was the strongest predictor of growth. Relative (proportional) growth rate declined steadily from the smallest to largest animals; linear growth rate increased until individuals reached intermediate size and then declined to zero at large size. A body mass twice as large as other individuals in the container was associated with somewhat lower growth rate. This latter result indicates reduced competitive ability in relatively large individuals, although the effect of relative size was much less important than that of absolute size. These results support the prediction that relatively large individuals enjoy a competitive advantage only when they can interfere with smaller individuals (Persson, 1985; Uchmanski, 1985; Weiner & Thomas, 1986). Our data also confirm the connection between individual growth and the size distribution of the group (Magnuson, 1962; Rubenstein, 1981; Ziemba & Collins, 1999). We observed that size distributions became less variable when composed of animals of mixed size, whereas variation did not change appreciably when the four tadpoles started out at the same size.

The decline in relative growth rate with increasing size agrees with Wilbur and Collins's (1973) observation that tadpole growth can be described as an exponential process that dampens exponentially, at least until the animal approaches metamorphosis (see also Peacor & Pfister, 2006). In other words, instantaneous relative growth rate declines as a tadpole increases in body size, much as it does in plants (Rees et al., 2010; Weiner & Thomas, 1986). Werner and Gilliam (1984) argue that the growth decline with size may explain the timing of ontogenetic habitat shifts in diverse organisms, including amphibians. Our results are also consistent with Werner's (1994) conclusion that smaller amphibian larvae become relatively more efficient under resource limitation. Indeed, the decline in absolute growth rate with increasing size relative to competitors in the same container—which was also nearly significant for relative growth rate—indicates that larger individuals perform less well in competition. In mixed‐size groups, smaller animals apparently obtained and assimilated more food, both relative to their body size (Figure 1a) and in absolute terms for some range of sizes (Figure 1b).

The predominant importance of absolute body size and the negative influence of relative size in this experiment differs from findings in many fish and other amphibians (Abbott & Dill, 1989; Brown, 1946; Karplus et al., 2000; Smith, 1990; Woodward, 1987). We believe that the explanation relates to the mechanism of interaction among competing individuals. Most comparable experiments on fish and amphibians created groups that interacted by interference competition (Abbott & Dill, 1989; Karplus et al., 2000; Smith, 1990; Ziemba & Collins, 1999). Models predict that relatively large individuals are favored under interference (Peacor et al., 2007a; Persson, 1985; Uchmanski, 1985). In our study, the animals almost certainly competed for resources, because interference mediated by *A. richardsi* (Wong et al., 2000) is unlikely in such a short experiment and exploitation is the predominant mechanism of competition in anurans under usual conditions (Biesterfeldt, Petranka, & Sherbondy, 1993; Laufer & Maneyro, 2008; Morin & Johnson, 1988; Petranka, 1989; Smith, 1983; Werner, 1992). Therefore, growth in our experiment should be related to rates of resource harvesting and energy expenditure; the allometry of these processes indicates that the net energy available for growth will frequently decline with body size (Brown et al., 2004; Peacor & Pfister, 2006; Werner, 1994). In fact, fish also show reduced growth with increasing relative body size when competition is mainly exploitative (Huss, Bystrom, & Persson, 2010).

Our results support a theoretical expectation that the tendency of a population of growing and interacting organisms to become more or less similar in body size depends on the way in which individuals interact. This suggests a mechanistic connection between the behavior of individual organisms and the size distribution of their group, a population‐level property with many ecological and evolutionary implications (Fordyce, 2006; Peacor et al., 2007b; Pfister & Stevens, 2002; Shelton et al., 2013). A proper test of this hypothesis awaits an experiment in which individual growth rate can be tied to a specific manipulation of the mechanism of competition. This is difficult to implement without influencing other elements of the interaction (e.g., Holdridge, Cuellar‐Gempeler, & TerHorst, 2016); one approach might establish combinations of above‐ and below‐ground barriers in certain kinds of plants (e.g., McPhee & Aarssen, 2001).

## Conflict of Interest

Nothing to declare.

## Author Contributions

JVB obtained funding and arranged permits, designed the study, analyzed the data, and wrote the manuscript. EC and JSH designed the study, collected the data, analyzed the data, and commented on the manuscript.

## Data Deposition

The data are available in an on‐line Appendix S1.
